# How is that? Knotting of a peripherally inserted central venous catheter

**DOI:** 10.4103/0019-5049.65367

**Published:** 2010

**Authors:** Gnanamuthu Birla Roy, Ajit A Cheriyan, Manbha L Rymbai

**Affiliations:** Department of Cardiothoracic Surgery, CMC Hospital, Vellore, Tamil Nadu, India

Sir,

An obese young man underwent a right posterolateral thoracotomy when the right basilic vein was cannulated in the conventional manner. A 14-G needle was used and a 75-cm 16-G peripherally inserted central venous catheter (PICC; Cavafix, B.Braun, Melsungen AG, Germany) was inserted. Some difficulty was encountered in accessing the vein, which was attributed to the patient’s obesity. The catheter was advanced without resistance and was fixed at a distance of 40 cm, the measured distance between the sternal notch and the puncture site. This line was used intraoperatively. During surgery, the catheter was incidentally spotted in the superior venacava. The tip, however, was not visualised since it was inside the right atrium. In the postoperative period, the fluids were not flowing freely through the catheter. Efforts to improve it by flushing and manipulating the catheter were unsuccessful. Hence, it was decided to remove the same.

**Figure 1:**
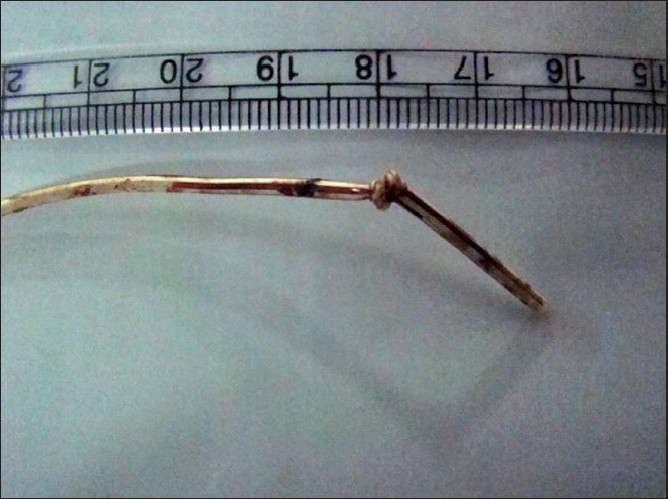
Peripherally inserted venous catheter with a knot about 2.0 cm from the tip

**Figure 2:**
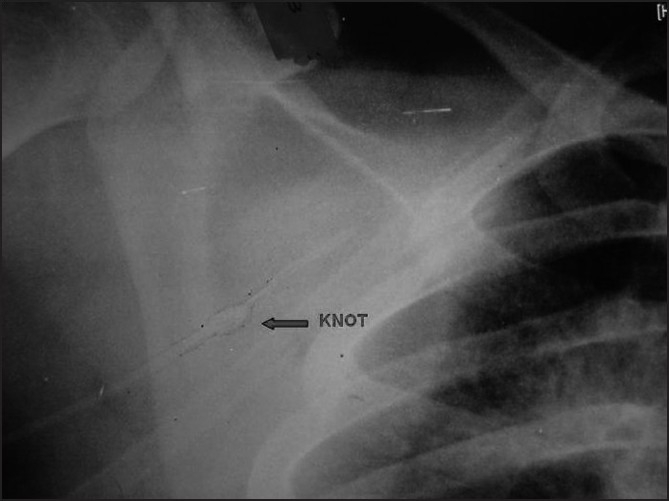
Magnified view of the immediate postoperative chest X-ray suggestive of a knot in the catheter identified by retrospective scrutiny

There was no resistance to the withdrawal till a distance of about 2.5 cm from the tip where it got impacted. Palpation just above the puncture site revealed a thickening in the catheter suggestive of a knot. With a venous tourniquet applied in the midarm to prevent a proximal migration of fragments in case of catheter fracture, steady traction was applied to the catheter to deliver it out. It revealed a well-formed knot about 2.0 cm from the tip [Figure 1]. Bleeding at the exit site was controlled with pressure. A retrospective scrutiny of the immediate postoperative chest X-ray showed a suspicious shadow in the catheter [Figure 2].

PICCs are routinely used to administer drugs and to monitor venous pressure. Common complications include phlebitis, thrombosis, malfunction, infections, bleeding, dislodgement and migration. Complications like cardiac arrhythmia and knotting are rarer.[1,2]

It has been postulated that knotting of the PICC lines after a basilic vein cannulation can happen either at the puncture site or at the brachio-cephalic venous junction.

Two mechanisms are suggested for this at the puncture site. One is that the cannula would have counterpunctured the vein to create an exit port for the catheter. The second is that the cannula would have slipped out of the vein during the advancement of the catheter causing the formation an extravascular knot.[3]

Abduction of the arm to 45° to straighten the axillary vein to prevent knotting at the brachio-cephalic junction which is at a distance of about 13–14 cm from the insertion site at the cubital fossa has been stressed.[4]

The exact reason for knot formation in our patient is uncertain. It could have happened due to a counterpuncture at the time of the difficult cannulation. Such mechanisms have been implicated even when there is no resistance during cannulation or catheter advancement.[3]

We assert that knot formation should be suspected whenever flow through a PICC is compromised, after the commoner causes are ruled out, especially if there had been difficulty in cannulation, catheter advancement or stillet removal. This should be confirmed by fluoroscopy or a chest X-ray and decannulation planned immediately.

Aggressive attempts at decannulation should be avoided to minimize damage to the vein or catheter fracture. Whenever possible, the knot should be brought to an area of easy surgical access and removed by venotomy. Using introducers to unravel the knot, a technique suggested for the stiffer catheters, is inappropriate for PICC since the knots here are tighter and the chances of breakage are higher.[5] When a knot is stuck inside a major vessel or the heart, interventional radiological techniques or a major surgical procedure will be indicated.[6]
